# Localization of ZIP14 and ZIP8 in HIBCPP Cells

**DOI:** 10.3390/brainsci10080534

**Published:** 2020-08-08

**Authors:** Shannon E. Morgan, Horst Schroten, Hiroshi Ishikawa, Ningning Zhao

**Affiliations:** 1 Department of Nutritional Sciences, University of Arizona, Tucson, AZ 85721, USA; morgans3@arizona.edu; 2 Department of Pediatrics, Pediatric Infectious Diseases, Medical Faculty Mannheim, Heidelberg University, 68167 Mannheim, Germany; horst.schroten@umm.de; 3 Department of Neurosurgery, University of Tsukuba, Tsukuba, Ibaraki 305-8575, Japan; ishi-hiro.crm@md.tsukuba.ac.jp

**Keywords:** ZIP14, ZIP8, Manganese, HIBCPP, Blood-cerebrospinal fluid barrier, SLC39A14, SLC39A8

## Abstract

The blood–cerebrospinal fluid barrier (BCB) is important in maintaining brain manganese (Mn) homeostasis. This barrier consists of a single layer of epithelial cells, connected by tight junctions, that restrict the passage of nutrients to only allow molecules to be carried through the membrane by a transporter. These epithelial cells are polarized with asymmetrical blood-facing and cerebrospinal fluid-facing sides. Here, we have established a polarized model of a human choroid plexus papilloma cell line, HIBCPP. For the first time, Mn importers ZIP14 and ZIP8 were identified in HIBCPP cells and were found to be enriched at the basolateral and apical sides of the cell monolayer, respectively. The localization of each ZIP protein adds to the understanding of Mn transport across the HIBCPP BCB model to help understand the mechanism of Mn homeostasis within the brain.

## 1. Introduction

The central nervous system is dependent on a controlled environment to function optimally. This environment is established through the passive and active transport mechanisms within the blood–brain barrier (BBB) and the blood–cerebrospinal fluid barrier (BCB). While the BBB is studied extensively for its role in immune factor transport [1] and ion/nutrient exchange [2], the BCB has more recently become a focus of study to understand the separation between the brain environment alongside the cerebrospinal fluid (CSF) and the systemic blood circulation [3].

The BCB exists at the choroid plexus, a single cell layer of choroid plexus epithelial cells that are connected to each other by tight junctions and anchored to a basement membrane surrounding blood vessels. It is found in all four ventricles of the brain. The choroid plexus is responsible for the secretion of CSF, as well as the exchange of various factors and nutrients between the CSF and the blood [4]. Models of the BCB include primary cultures of choroid plexus epithelial cells isolated from postmortem human tissue, rodents, or larger mammals. Differences in species and cell culture protocols make it difficult to compare the data between individual studies. A reliable in vitro model that uses an immortalized and widely available cell line would help to standardize the experiments and results surrounding BCB research. HIBCPP cells have recently been identified as a consistent model of the BCB, and several publications have utilized these cells as a barrier model [5,6,7]. The HIBCPP cell line was established in 2005 from a human choroid plexus papilloma [8] and has been characterized in several studies that indicate that HIBCPP cells have many critical functional characteristics of choroid plexus epithelial cells—namely, the ability to polarize and express marker proteins to match choroid plexus epithelial cells in vivo [5,6,9].

However, previously unreported are the characteristics of HIBCPP cells relating to manganese (Mn) metabolism. As an essential nutrient, Mn is present in both the blood and CSF [10] and is required for the normal function of enzymes in choroid plexus epithelial cells [11]. One in vitro study on the BCB and BBB reveals that the BCB, not the BBB, is more sensitive to high blood Mn levels and is likely to be the primary site of Mn entry into the brain [12]. While several metal transport proteins are expressed on choroid plexus cells, none have been identified as Mn transporters [12,13]. Furthermore, it remains unknown which Mn transporters are expressed in HIBCPP cells.

Recently, it has been identified that members of the ZIP family proteins, ZIP14 and ZIP8, are two major Mn importers expressed in the epithelial cells of the liver and intestine [14,15,16,17]. ZIP14 imports Mn from blood into enterocytes and hepatocytes within the intestine and liver, respectively, while ZIP8 imports Mn from the bile to hepatocytes. In patients with ZIP14 deficiency, high blood Mn and the accumulation of Mn in the brain result in irreversible neurodegeneration associated with motor disabilities [18,19]. The Mn overload in individuals lacking ZIP14 is likely caused by a combined effect of increased intestinal Mn absorption and decreased hepatobiliary Mn excretion [14,15,20,21]. Patients with ZIP8 deficiency have blood Mn levels below detectable limits and present with severe psychomotor and intellectual disabilities [22,23,24], which are due to severe Mn deficiency caused by decreased Mn reabsorption from the bile [17].

The functions of ZIP14 and ZIP8 as Mn importers have been well established through in vitro studies. In hepatic HepaRG and WIF-B cells, ZIP14 was expressed at the basolateral membrane and imported Mn in a time- and temperature-dependent manner based on the Mn uptake assay [25,26]. In *Xenopus* oocytes, Mn uptake in cells expressing murine ZIP14 protein was significantly higher than that in control oocytes, indicating the role of ZIP14 in mediating Mn import [27]. In enterocyte-like Caco-2 cells, ZIP14 was primarily detected on the basolateral membrane, and it was determined to account for over 95% of the Mn uptake from the basolateral side [21]. In kidney proximal tubule cells, the knockdown of either ZIP14 or ZIP8 was associated with a significant decrease in Mn uptake [28]. In mouse fibroblasts (MEF), ZIP8 overexpression resulted in elevated cellular Mn accumulation; moreover, in MEF cells, ZIP8 had a higher affinity for Mn than all other trace metals tested, except for cadmium [29]. In HEK293 cells, ZIP8 was detected on the plasma membrane and ZIP8 overexpression increased cellular Mn accumulation [30]. Together, these studies clearly demonstrate that both ZIP14 and ZIP8 are Mn importers.

Similarly to the epithelial cells of the intestine and liver, HIBCPP cells form a polarized monolayer with specific apical and basolateral orientation [5,31,32]. Identifying Mn transporters in these cells will help us to understand the mechanisms of Mn homeostasis within the brain. Whether ZIP14 and ZIP8 are expressed in HIBCPP cells and whether these two Mn transporters are localized to apical or basolateral plasma membranes are not known. In the present study, we identified that both ZIP14 and ZIP8 are expressed in HIBCPP cells and that both transporters are involved in cellular Mn uptake. In addition, we found that ZIP14 and ZIP8 are enriched at opposite sides of HIBCPP cells. These results provide novel insights for the understanding of potential Mn transport mechanisms in HIBCPP cells.

## 2. Materials and Methods

### 2.1. Cell Culture

HIBCPP cells were maintained in DMEM/F12 (Corning Inc., Corning, NY, USA) supplemented with 10% fetal bovine serum, 100 µg/mL streptomycin, 100 units/mL penicillin, 4 mM L-glutamine, and 5 µg/mL insulin. To be consistent with previously published results using HIBCPP cells, only cells before passage 37 were used for experiments [33]. HEK293 and A549 cells were cultured in DMEM supplemented with 10% fetal bovine serum, 100 µg/mL streptomycin, and 100 units/mL penicillin. All cells were grown in a humidified incubator at 37 °C with 5% CO_2_. Media was changed every 2–3 days and trypsin (0.05% in phosphate buffered saline [PBS]) (Life Technologies, Grand Island, NY, USA) or Accutase (Corning Inc., Corning, NY, USA) was used to detach cells for passaging.

### 2.2. RNA Isolation, PCR, Quantitative Real-Time PCR (qRT-PCR), and Melting Curve Analysis

Total RNA was isolated from HIBCPP cells using the Quick-RNA MiniPrep Plus kit (Zymo, Irvine, CA, USA). Complementary DNA was synthesized from isolated RNA using M-MuLV Reverse Transcriptase (New England BioLabs, Ipswich, MA, USA). To verify the primers, PCR was performed using a Direct PCR kit (Bimake, Houston, TX, USA) and a thermal cycler (C1000 Touch Thermal Cycler, Bio-Rad, Hercules, CA, USA). The primers used were as follows: *ZIP8*-Forward 5′-TGG TTG CAC CCC TCA CAA AT-3′, reverse 5′-CAC ATG GTG CAC TGA AAC CG-3′; *ZIP14*-forward 5′-GTC TGG CCT TTG GCA TCC T -3′, reverse 5′-AGG GAA CAT ATC AGC CAG AGA AAT AG-3′. The PCR products were loaded onto a 2% agarose gel, separated by electrophoresis, and purified with a Wizard SV Gel and PCR Clean-up System (Promega, Madison, WI, USA). The purified PCR products of the primers for ZIP8 or ZIP14 were further used as standards to quantify copy numbers of ZIP8 or ZIP14 mRNA by qRT-PCR analysis. The qRT-PCR procedure was performed using a SYBR Green PCR Master mix (ThermoFisher Scientific, Waltham, MA, USA) with the Applied Biosystems QuantStudio 5 system (ThermoFisher Scientific, Waltham, MA, USA). Copy numbers of ZIP8 and ZIP14 mRNA were determined by comparing Ct values with those obtained from standard curves as previously described [34]. The fluorescence signal from SYBR green intercalating dye was measured as the temperature increased in each cycle to determine the melting point of each DNA product. The melting point is determined by the base pair composition in a strand of double-stranded DNA. For each suitable primer set, only one DNA product should be copied, resulting in a single peak at one melting point. Thus, the melting curve results were analyzed to confirm the presence of single peaks for each primer set.

### 2.3. siRNA and Plasmid Transfections

HIBCPP cells were added to 6-well dishes at 3.5–4.5 × 10^5^ cells per well. The next day, siRNA targeting either ZIP8 (5′-CCC AAA CUG UCA GAA AUA GGG ACG A-3′) (Origene Technologies Rockville, MD, USA; Cat no. SR11962B) or ZIP14 (5′-CCC UCU GGA AGA UUA UUA UGU CUC C-3′) (Origene, Cat no. SR308328B) was transfected into HIBCPP cells using the Lipofectamine RNAiMAX transfection reagent (ThermoFisher Scientific, Waltham, MA, USA). A scramble siRNA was used as the negative control. The transfection procedure was completed per the manufacturer’s protocol using 60 pmol of siRNA. When transfecting HEK293 or A549 cells, 4 × 10^5^ cells per well were seeded, and transfection occurred using 30 pmol of siRNA.

To overexpress FLAG-tagged proteins in HEK293 cells, pCMV-Entry-hZIP14-myc-FLAG or pCMV6-Entry-hZIP8-myc-FLAG plasmid (Origene, Rockville, MD, USA) was transfected into cells using an Effectene transfection reagent (Qiagen, Hilden, Germany). To knock down the expression of FLAG-tagged proteins, ZIP14-targeting or ZIP8-targeting siRNA was co-transfected one hour after the plasmid transfection. Cell lysates were collected 24 h after the co-transfection.

### 2.4. Transwell Culture

For all biotinylation experiments, cells were grown on Transwell inserts (24 mm diameter, 0.4 µm pore size, polystyrene) (Corning Inc.). HIBCPP cells were lifted with 0.05% trypsin or Accutase for 15 min at 37 °C, followed by manual dissociation with a 1 mL pipette. Growth media was added to stop trypsinization or Accutase digestion, and viable cells were counted using Trypan blue (VWR, Radnor, PA, USA). Then, 1.2 × 10^6^ cells were added to the upper chamber of a 6-well Transwell insert and media was added so that the total volume was 1.6 mL in the upper chamber only. The day after seeding, non-adhered cells were removed and growth media was changed in the upper chamber, and 2.5 mL of growth media was added to the lower compartment. Every 2–3 days thereafter, media was aspirated, cells were washed once with fresh media, and then fresh media was added to the upper and lower chambers of each well. Cells were used for biotinylation experiments when they reached confluence, which was typically 7 days after seeding.

### 2.5. Measurement of Transepithelial Electrical Resistance (TEER)

For TEER measurements, 4 × 10^5^ cells per well were seeded in 12-well Transwells as described earlier in this section. The media levels in the upper and lower chambers were 0.8 mL and 1.5 mL, respectively. EVOM^2^ with STX2 electrodes (World Precision Instruments, Sarasota, FL, USA) was used to measure the resistance across the cell layer. The resistance from a single well containing media only (control resistance) was measured daily to calculate TEER (TEER = [Experimental Resistance – Control Resistance] × Transwell area) [5,6]. Resistance was measured from three areas within each well, and the average was used to calculate the TEER for each well. Measurements were taken daily for a minimum of 11 days.

### 2.6. Cell Surface Protein Biotinylation

Surface biotinylation was performed on HIBCPP cell monolayers grown on 6-well Transwell inserts. After removing growth medium, cells were washed twice with PBS^+/+^ (PBS containing 1 mM MgCl_2_ and 0.1 mM CaCl_2_) pre-warmed to 37 °C, then twice more with pre-chilled PBS^+/+^ before the insert was transferred to a new 6-well plate. Basolateral or apical membrane proteins were biotinylated by adding 0.5 mg/mL Sulfo-NHS-SS-biotin (ThermoFisher Scientific, Waltham, MA, USA) in PBS^+/+^ to either the basolateral or apical side. Cells were incubated at 4 °C for 30 min with gentle agitation. For each cell monolayer, PBS^+/+^ was added to the opposite side during incubation. Then, cells were washed twice with 4 °C Tris buffered saline (TBS) before quenching with 100 mM glycine for 20 min at 4 °C with gentle agitation. Glycine was removed, and the quenching step was repeated once more, followed by two more 4 °C TBS washes. The insert was excised and transferred to a new 6-well plate. Cells were lysed with 1 mL of NETT buffer (150 mM NaCl, 5 mM EDTA, 10 mM Tris, 1% Triton X-100, and 1× protease inhibitor mixture (Bimake, Houston, TX, USA), pH 7.4) and lysate was collected in 1.5 mL collection tubes. The lysate was chilled on ice for 20 min, vortexed every 5 min, and centrifuged at 10,000× *g* for 10 min. The supernatant containing the “whole” lysate was transferred to 1.5 mL collection tubes. An aliquot of 300 µL was stored at −80 °C for future analysis, while the remaining lysate was transferred to a Pierce centrifuge column pre-loaded with 100 µL of Pierce high capacity NeutrAvidin agarose beads (ThermoFisher Scientific, Waltham, MA, USA), per the manufacturer’s protocol. Samples were incubated for 2 h at 4 °C on a rocker. The column was centrifuged for 1 minute at 1500× *g* to collect the flow-through. The beads were washed three times with TBS containing protease inhibitors. After each wash, the column was centrifuged for 1 minute to remove the flow through. To elute the biotinylated proteins, 100 µL of 1× sample buffer (1.7% (*w*/*v*) SDS, 5% (*v*/*v*) glycerol, 150 mM Dithiothreitol [DTT], 58 mM Tris, pH 6.8) was added to the agarose beads. Then, the samples were incubated for 1 hour at room temperature, with hand mixing every 10 min. The columns were centrifuged for 2 min at 1500× *g* to collect the surface protein fraction. This membrane protein fraction was stored at −80 °C for further Western blot analysis.

### 2.7. Manganese Uptake

^54^Mn uptake was measured in HIBCPP cells grown on 6-well plates for 72 h. The uptake procedure was performed as previously described [21]. Briefly, two-times concentrated (0.2 μM) ^54^Mn solutions were prepared from ^54^MnCl_2_ (PerkinElmer Inc., Waltham, MA, USA) complexed to citrate on the day of the experiment. Higher concentrations of Mn were achieved by adding Mn citrate in the required amounts. During the experiments, cells were washed three times with PBS^+/+^ and pre-incubated in 1 mL of transport medium (DMEM with 1 mM pyruvate and 20 mM HEPES, pH 7.4) at 37 °C (or 4 °C) in a humidified incubator (or on ice) for 30 min. ^54^Mn uptake experiments were initiated by adding 1 mL of two-times concentrated ^54^Mn solutions, and the cells were incubated at 37 °C (or 4 °C) for 5 min. The uptake procedure was stopped by removing the media and washing the cells five times with ice-cold washing buffer (PBS with 1 mM EDTA). The cells were lysed in 1 mL 0.5 M NaOH, and 100 μL of lysate was used to determine the protein concentration. An aliquot of 400 μL of the lysate was used to quantify the cellular content of ^54^Mn by gamma counting (WIZARD^2^ Automatic Gamma Counter, PerkinElmer Life Sciences).

### 2.8. Immunocytochemistry and Confocal Microscopy

HIBCPP cell monolayers grown on Transwell inserts were fixed and permeabilized with methanol at minus 20 °C, followed by pre-chilled acetone. After inserts were excised and placed in a new 6-well plate, cells were blocked with blocking buffer (1% bovine serum albumin in PBS^+/+^) for 2 h at room temperature. After blocking, cells were incubated overnight in blocking buffer with rabbit anti-ZO-1 antibody (Proteintech, Rosemont, IL, USA) (1:100) in a humidified chamber at 4 °C, followed by a 2-h incubation with Alexa Fluor-488 conjugated goat anti-rabbit antibody (Life Technologies, Carlsbad, CA, USA) (1:500) at room temperature. Cells were treated with 4′,6-diamidino-2-phenylindole (DAPI) (1 µg/mL PBS) for 5 min at room temperature for nuclear staining. Membranes with cells were mounted on glass slides in a ProLong Diamond Antifade Mountant (ThermoFisher Scientific, Waltham, MA, USA) beneath a glass coverslip. Images were obtained using a Zeiss LSM880 inverted confocal microscope with a 20× objective at the Marley Imaging Core within the University of Arizona (Tucson, AZ, USA).

### 2.9. Western Blotting Analysis

Western blotting analysis was completed as previously described [21]. Cells in lysis buffer were thoroughly vortexed, followed by centrifugation 10,000× *g* to separate cell nuclei and larger debris. Protein quantification of clarified supernatant was determined using the RC DC protein assay kit (Bio-rad, Hercules, CA, USA). Equal amounts of protein samples were prepared with 1x Laemmli buffer, heated at 37 °C for 30 min, then loaded into wells of 7.5–10% polyacrylamide SDS gels. Proteins were electrophoretically separated at 120V for 60–90 min. Proteins were transferred to nitrocellulose membranes for 2 h at 100V. All blots were blocked in blocking buffer consisting of 5% nonfat milk in TBS with 0.1% Tween-20 (TBST) for one hour and then probed with anti-human ZIP8 (Proteintech, Rosemont, IL, USA) (1:2000), ZIP14 (1:1000), MRP-1 (Cell signaling Technology, Danvers, MA, USA) (1:1000), or divalent metal transporter-1 (DMT-1) (Proteintech, Rosemont, IL, USA) (1:1000) primary antibodies, followed by anti-rabbit-HRP secondary antibody (Millipore Sigma, Burlington, MA, USA) (1:4000). Horseradish peroxidase (HRP)-conjugated GAPDH or Beta-Actin antibodies (Proteintech, Rosemont, IL, USA) (1:20,000) were used to probe for normalization proteins. The ZIP14 antibody was made in house as previously described in detail [21]. Blots were imaged with the ChemiDoc MP imaging system and Image Lab software (Bio-Rad Bio-Rad, Hercules, CA, USA).

### 2.10. Statistical Analysis

Results were analyzed using PRISM 5 software (GraphPad, La Jolla, CA, USA). Three sets of data were analyzed by one-way ANOVA. Tukey’s post hoc comparisons tests were performed for multiple comparisons. Two sets of data were analyzed by the Student’s *t*-test. A *p*-value < 0.05 was considered statistically significant.

## 3. Results

### 3.1. The Detection of ZIP14 and ZIP8 mRNAs in HIBCPP Cells

**Figure 1:**
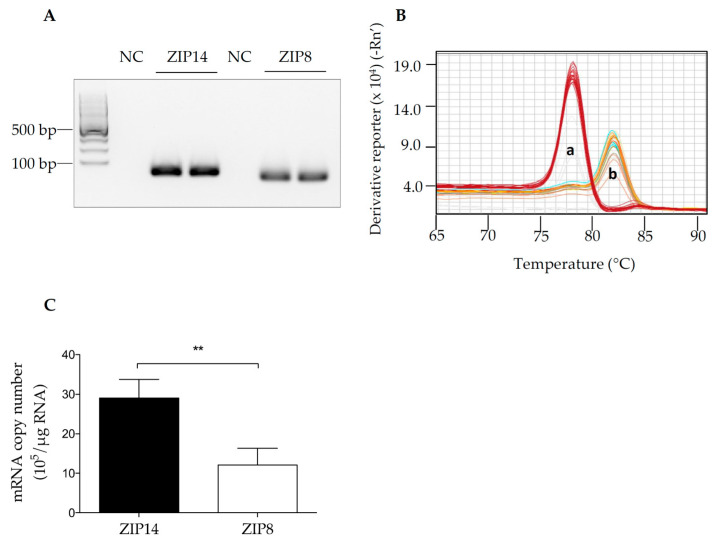
mRNA levels of ZIP14 and ZIP8 can be detected in human choroid plexus papilloma cell line (HIBCPP cells). (**A**) PCR products of each primer set were loaded onto a 2% agarose gel, revealing a single amplicon band for each primer set. Predicted sizes: 104 bp (*ZIP14*) and 74 bp (*ZIP8*). NC: Non-template control. (**B**) Melt curve plots show individual peaks for each primer set. a: *ZIP8*, b: *ZIP14*. (**C**) Total RNA was isolated from untreated HIBCPP cells grown for 72 h. Copy numbers were determined by qRT-PCR and revealed a higher expression of *ZIP14* compared to *ZIP8*. Data represent three independent experiments. ** *p* < 0.01.

To determine whether *ZIP14* and *ZIP8* are expressed in HIBCPP cells, we first aimed to use qRT-PCR analysis to measure the expression levels of these two genes. The specificity of qRT-PCR primer sets was evaluated by DNA gel electrophoresis and melt curve analysis to ensure the validity of the results presented in our studies. The correct sizes of PCR amplification products and absence of non-specific amplification were verified by agarose gel electrophoresis (Figure 1A). Additionally, for primer sets targeting *ZIP14* and *ZIP8*, one clear peak for each gene was present in the melt curve analysis, indicating that a single amplicon was obtained in the RT-PCR procedure (Figure 1B).

Both ZIP14 and ZIP8 are ubiquitously expressed [35,36,37]. Tissues with high levels of ZIP14 include the liver and small intestine [35,36,38]; while ZIP8 is abundantly detected in the lung and large intestine [36]. However, the expression levels of ZIP8 and ZIP14 in the choroid plexus or choroid plexus-derived cells have not been reported. As a cell line derived from a human choroid plexus papilloma, HIBCPP cells were broadly used as a model for choroid plexus epithelium [6,9,33,39]. Here, for the first time, we reported that both *ZIP14* and *ZIP8* are expressed in HIBCPP cells. Using a standard curve and comparing Ct values between samples and standards, we were able to determine the copy number of each gene. *ZIP14* expression was about 2.3 times higher compared to *ZIP8* in HIBCPP cells (Figure 1C).

### 3.2. Both ZIP14 and ZIP8 Proteins Are Present in HIBCPP Cells

**Figure 2:**
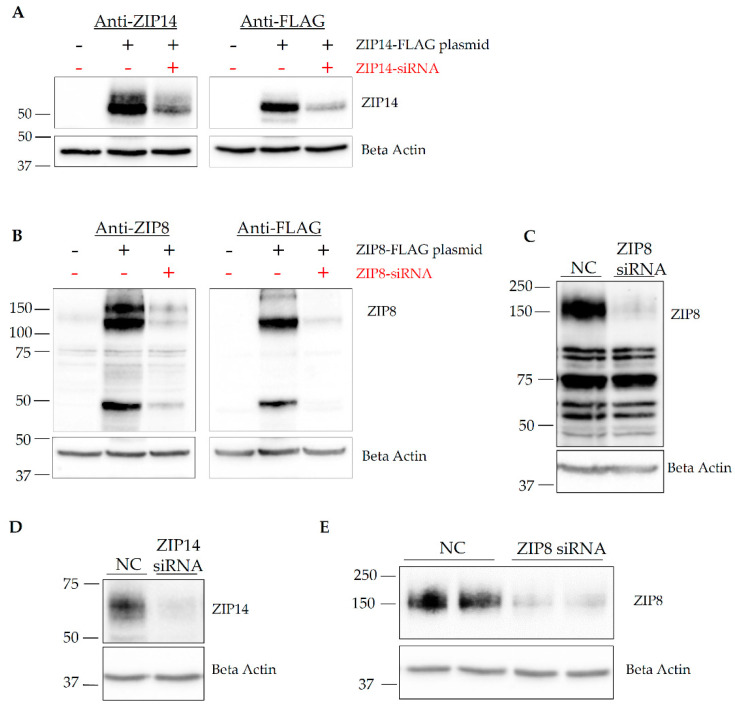
Validation of anti-ZIP14 and anti-ZIP8 antibodies and the detection of ZIP14 and ZIP8 proteins in HIBCPP cells. (**A**) HEK293 cells were transfected with a control empty vector (−), or a vector expressing FLAG-tagged ZIP14 (+). In the meantime, cells were transfected with negative control siRNA (−) or ZIP14-targeting siRNA (+). Antibodies used to detect ZIP14 and FLAG identify the FLAG-tagged protein at approximately 55 kDa. (**B**) HEK293 cells were transfected with the control vector (−) or a vector expressing FLAG-tagged ZIP8 (+). Concurrently, cells were transfected with negative control siRNA (−) or ZIP8-targeting siRNA (+). (**C**) siRNA knockdown of endogenous ZIP8 in A549 cells confirmed that the ZIP8 antibody detects the endogenous transporter at 150 kDa. In HIBCPP cells, ZIP14 (**D**) and ZIP8 (**E**) proteins were both detected. siRNA knockdown confirmed the identities of these proteins. The plasmid or siRNA transfection was carried out for 24 h (**A**,**B**) or 48 h (**C**–**E**) prior to Western blot analyses using anti-ZIP14, anti-ZIP8, or anti-FLAG antibodies. Beta Actin was used as a loading control.

Since the presence of RNA does not necessarily reflect actual expression of the protein of interest [40,41], we next asked the question of whether ZIP14 and ZIP8 proteins could be detected in HIBCPP cells. To investigate the presence of ZIP14 and ZIP8 proteins, we first tested the specificity of the antibodies used for Western blotting analysis. As of this publication, there are no commercially available human ZIP14 antibodies for Western blotting, so one has been generated in our laboratory. Additionally, commercially available human ZIP8 antibodies seem to vary drastically. To determine the specificity of the ZIP14 antibody, we transiently transfected HEK293 cells with a plasmid encoding FLAG-tagged ZIP14. After plasmid transfections, cells were transfected with either negative control (NC) or ZIP14-targeting siRNA. Results indicate the presence of the ZIP14 band at the predicted molecular weight of 55 kDa, confirmed by both the anti-ZIP14 antibody and anti-FLAG antibody. Additionally, siRNA transfected cells have a marked reduction in ZIP14 expression detected by both anti-ZIP14 and FLAG antibodies (Figure 2A and Figure S1A).

To verify the anti-ZIP8 antibody and ZIP8-specific siRNA, a similar procedure was carried out in HEK293 cells using a plasmid encoding FLAG-tagged ZIP8 and ZIP8-targeting siRNA. Results indicated the presence of ZIP8 bands at around 50 kDa, as well as at approximately 100 kDa and 150 kDa positions (Figure 2B and Figure S1B). The predicted molecular weight of ZIP8 is approximately 50 kDa; therefore, the higher molecular bands detected by Western blotting could represent multimer forms of ZIP8 [30,42,43]. To determine the signal pattern of endogenous ZIP8, we performed another siRNA knockdown in A549 cells, which are alveolar epithelial cells expressing high levels of endogenous ZIP8 protein [44]. In A549 cells, endogenous ZIP8 was only detected at the 150 kDa position (Figure 2C and Figure S1C). Together, these experiments carefully validated both anti-ZIP14 and anti-ZIP8 antibodies, as well as their targeting siRNAs.

To determine whether ZIP14 and ZIP8 proteins are expressed in HIBCPP cells, we transfected HIBCPP cells with siRNA to knockdown either ZIP14 or ZIP8. Western blotting confirmed that both ZIP14 and ZIP8 proteins are present in HIBCPP cells (Figure 2D,E and Figure S1D,E).

### 3.3. Both ZIP14 and ZIP8 Contribute to Mn Accumulation in HIBCPP Cells

The BCB is an important part of the homeostatic infrastructure of the brain and is involved in the active transport of nutrients [45]. HIBCPP cells are frequently used to model the BCB, but Mn accumulation by HIBCPP cells has not been investigated. While the detection of ZIP14 and ZIP8 proteins in HIBCPP cells is novel, the activity of these transporters is unknown in this cell model. Our next goal was to confirm the activity of ZIP14 and ZIP8 as Mn transporters in HIBCPP cells.

**Figure 3:**
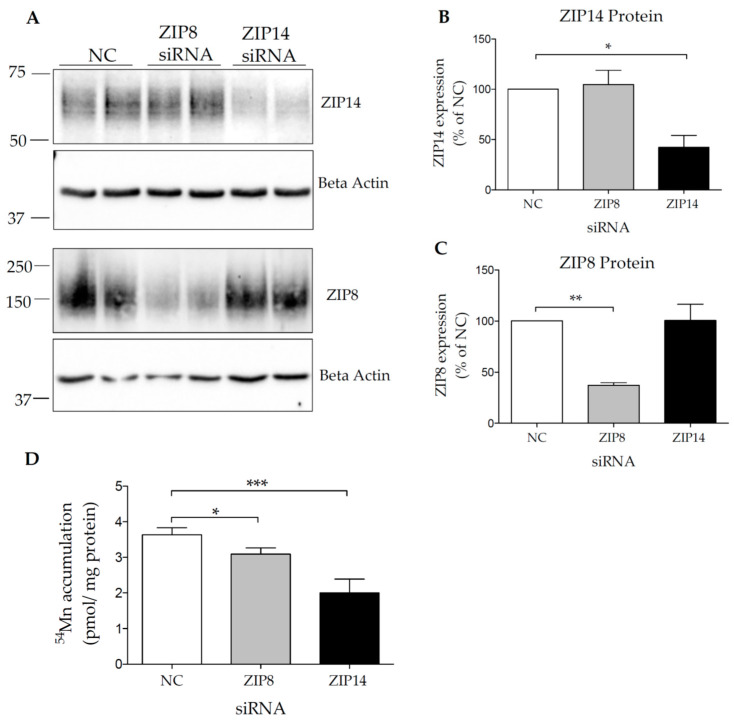
ZIP14 and ZIP8 are involved in Mn uptake in HIBCPP cells. (**A**) HIBCPP cells were transfected with either ZIP14, ZIP8, or negative control (NC) siRNA for 48 h, and lysates were probed for ZIP14 (upper) and ZIP8 (lower) proteins. Quantification of (**B**) ZIP14 and (**C**) ZIP8 protein levels. (**D**) HIBCPP cells were transfected with NC, ZIP14-, or ZIP8-specific siRNA for 48 h. Cells were incubated with 0.1 µM of ^54^Mn at 37 °C for 5 min. Cell-associated radioactivity was determined by gamma counting. The data are presented as means ± S.D. from four independent experiments. * *p* < 0.05, ** *p* < 0.01 and *** *p* < 0.001.

To determine whether ZIP8 and ZIP14 contribute to Mn uptake, HIBCPP cells were transfected with either negative control (NC), ZIP14-, or ZIP8-targeting siRNA. A representative blot for ZIP14 and ZIP8 knockdown is shown in Figure 3A. The knockdown efficiency of either ZIP14 or ZIP8 is close to 50%, with no significant change in ZIP14 expression after ZIP8 knockdown or no change in ZIP8 expression after ZIP14 knockdown (Figure 3B,C). HIBCPP cells transfected with ZIP14 siRNA contained approximately 44% less ^54^Mn than the control cells, while ZIP8 siRNA treatment resulted in about 14% less ^54^Mn uptake compared to the controls (Figure 3D) indicating that both ZIP14 and ZIP8 contribute to Mn uptake in HIBCPP cells. Due to the incomplete siRNA-mediated knockdown of each protein, we could not conclude that ZIP14 and ZIP8 are the only two transporters responsible for Mn uptake in HIBCPP cells. Future investigation should establish a stable knockout cell line to determine both transporters’ precise contribution to Mn uptake in these cells.

**Figure 4:**
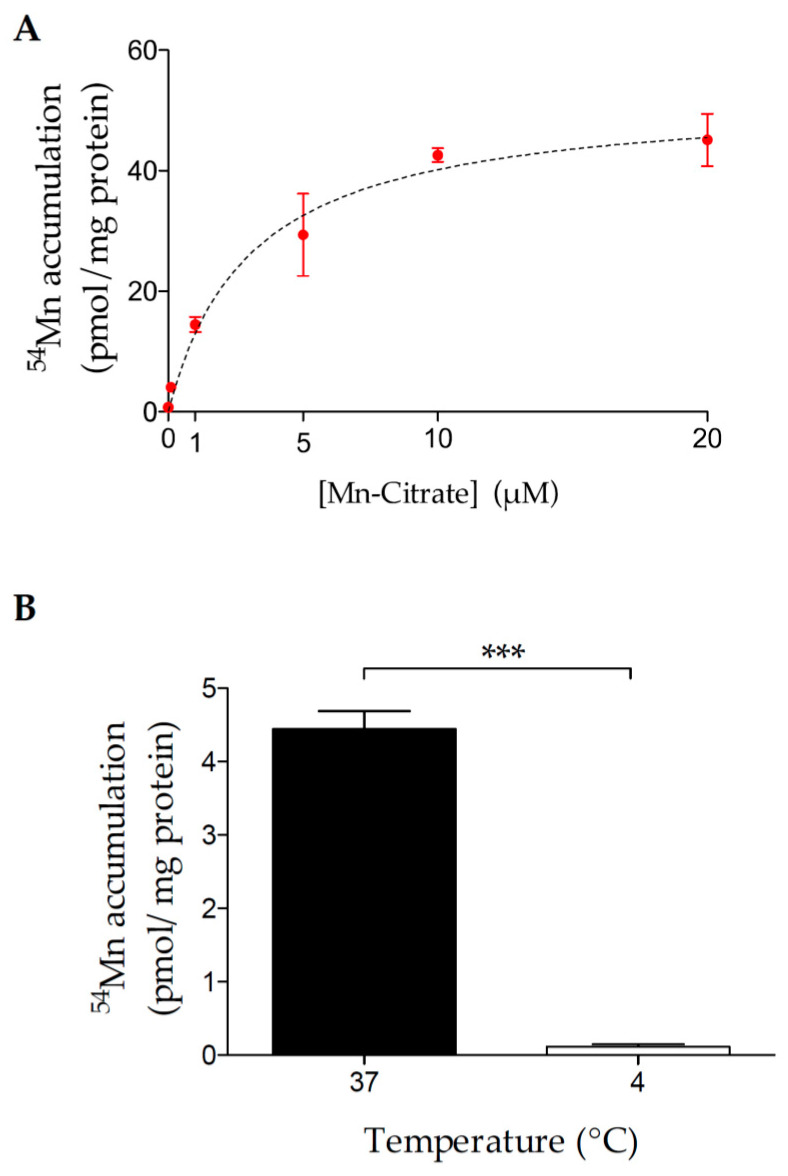
Characterization of Mn accumulation by HIBCPP cells. (**A**) Concentration-dependence of ^54^Mn uptake of HIBCPP cells. HIBCPP cells were grown on six-well plates for 48 h. Cells were incubated with uptake media containing different concentrations of ^54^Mn-Citrate for 5 min. (**B**) Temperature-dependence of ^54^Mn accumulation. Cells were incubated with 0.1 µM of ^54^Mn at 37 °C or 4 °C for 5 min. Cell-associated radioactivity was determined by gamma counting. The data are presented as means ± S.D. from three independent experiments. *** *p* < 0.001.

To confirm that Mn accumulation by HIBCPP cells is mediated by an active transport mechanism, we further characterized Mn uptake. The active transport process facilitates the movement of a substrate across the membrane relative to the amount of substrate until the transporter is saturated [46]. Additionally, active transport processes proceed optimally at temperatures close to 37 °C and are essentially blocked at 4 °C [46]. These two characteristics, concentration- and temperature-dependence, were investigated in our study to determine whether Mn enters HIBCPP cells via an active process. We measured ^54^Mn accumulation using a wide range of Mn concentration. Our results indicated that Mn accumulation by HIBCPP cells is a saturable biological process (Figure 4A). Moreover, we found that the incubation of HIBCPP cells at 4 °C almost completely abolished ^54^Mn uptake (Figure 4B). These results suggest that Mn accumulation by HIBCPP cells is mediated through an active transport mechanism.

### 3.4. Establishment of HIBCPP Transwell Cultures

**Figure 5:**
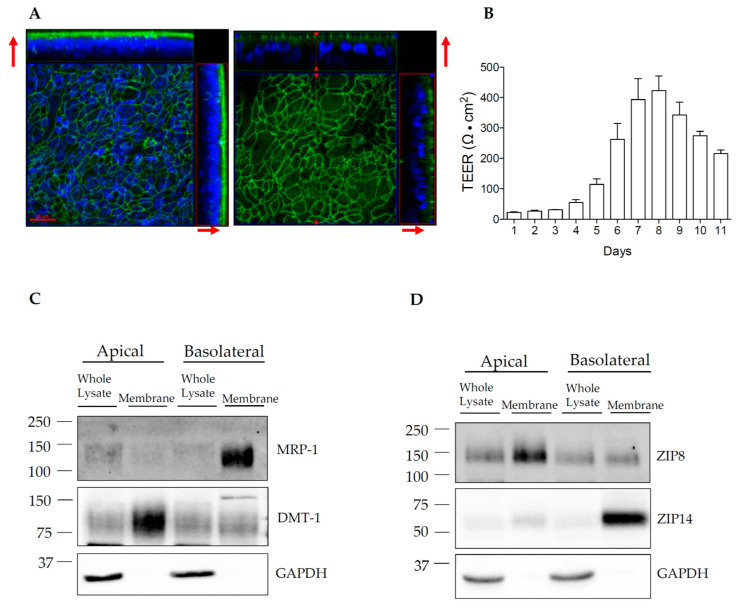
HIBCPP cells form a polarized monolayer connected by tight junctions. After seeding on a Transwell insert, HIBCPP cells grow to confluence. (**A**) After 7 days in culture, immunofluorescent antibody for ZO-1 (green) reveals the formation of cell–cell junctions. Nuclei are marked by 4′,6-diamidino-2-phenylindole (DAPI) (blue). Left image is a maximum intensity projection, right image represents a single z-stack. Red arrows point to the apical side of the cell layer. Scale bar: 20 µm. (**B**) HIBCPP monolayer integrity was monitored by Transepithelial Electrical Resistance (TEER) measurement (Days post-seeding are indicated on the X-axis). The data are presented as means ± S.D. from four independent Transwell cultures. (**C**) MRP-1, a known basolateral transporter in choroid plexus tissue, and DMT-1, an apical protein, were probed as basolateral and apical markers, respectively. (**D**) ZIP14 and ZIP8 are expressed on opposite sides of the membrane in a polarized HIBCPP cell culture. GAPDH is not enriched in the membrane fraction after biotinylation and therefore appears to only be present in the whole lysate.

In polarized epithelial cells, the apical and basolateral plasma membrane domains contain different nutrient transport proteins and carry distinct transport processes [47]. To determine the relative localization of ZIP14 and ZIP8 in HIBCPP cells, we aimed to use the Transwell culture system and cell surface protein biotinylation approach. This approach has been widely used to study the steady-state distributions of plasma membrane proteins in polarized epithelial cells [21,48,49]. We confirmed the expression of tight junction protein zonula occludens (ZO-1) in HIBCPP cells (Figure 5A). In addition, we evaluated the cell monolayer integrity by measuring the electrical resistance across the Transwell culture. HIBCPP cells are reported to have very low permeability at TEER values over 150 Ω · cm^2^ [5] to 400 Ω · cm^2^ [6]. Our measurements reveal TEER values at or approaching 400 Ω · cm^2^ within 7 to 8 days after seeding (Figure 5B). Therefore, biotinylation experiments are conducted when cells have visibly formed a monolayer after 7–8 days of growth. We first confirmed the polarity of the HIBCPP monolayer using Western blotting to probe for well-characterized apical or basolateral marker proteins. For the apical marker protein, we used divalent metal transporter-1 (DMT-1), which is mainly present on the apical side of polarized Caco-2 epithelial cells [21] and kidney proximal tubule epithelial cells [50]; for the basolateral marker protein, we used the multidrug resistance-associated protein 1 (MRP-1), since MRP-1 is expressed on the basolateral membrane of epithelial cells in choroid plexus tissue and in HIBCPP cells [5,51]. After biotinylation, it was confirmed that DMT-1 was highly enriched on the apical membrane of HIBCPP cells. In contrast, MRP-1 was detected mainly on the basolateral membrane (Figure 5C and Figure S3A).

### 3.5. ZIP14 and ZIP8 Are Expressed On Opposite Sides of Polarized HIBCPP Cells

Mn is present in both the blood and CSF, suggesting that the choroid plexus epithelial cells could take up Mn from both its apical and basolateral membrane domains. ZIP14 and ZIP8 are known to be critical for importing Mn into cells [17,21], and experiments with either transporter overexpressed resulted in an increase in Mn uptake [27,29,30]. In this study, we have found that both ZIP14 and ZIP8 play a role in Mn accumulation by HIBCPP cells. Both transporters are reported to have either basolateral or apical expression in other cell lines [17,21], but the distributions of ZIP14 and ZIP8 in choroid plexus-derived cells have not been reported. To determine the membrane localization of ZIP14 and ZIP8 in HIBCPP cells, we seeded the cells on Transwell inserts to allow the cells to form polarized monolayers. We found that ZIP14 and ZIP8 differ in their relative distributions: ZIP14 is enriched on the basolateral membrane (Figure 5D and Figure S3B), while ZIP8 is enriched on the apical side of HIBCPP cells (Figure 5D and Figure S3B). GAPDH, an intracellular protein, was used to confirm the lack of enrichment of cytosolic proteins in the membrane fractions of our samples. To our knowledge, this study is the first to report the presence of polarized Mn transport proteins in HIBCPP cells.

## 4. Discussion

A growing body of research regarding the choroid plexus as the site for BCB has used mammalian primary cultures of porcine [52,53] and rodent [54,55] choroid plexus tissues. While primary cultures are suitable to study choroid plexus epithelial cells for individual experiments, protocols and results from primary cultures of choroid plexus cells are difficult to compare, and the establishment of primary cultures from rodents requires a large number of animals to yield a small quantity of healthy epithelial cells [54,56]. Therefore, there is a need for a consistent and reliable in vitro model for the choroid plexus epithelial cells. HIBCPP cells were isolated from a human papilloma and continuously proliferate [8], making them more convenient and economical to use than primary cells. Particularly important as a model of the BCB, HIBCPP cells consistently polarize and are connected by tight junctions, which allows them to form an epithelial monolayer.

As an essential nutrient, Mn is required for normal brain function. It has been demonstrated that Mn enters the brain primarily through the BCB [12]. To investigate how Mn homeostasis is regulated within the BCB, we first need to identify which transporters are expressed in the choroid plexus epithelial cells. In this study, by using the HIBCPP cells as a model for choroid plexus epithelium, we identified that Mn importers ZIP14 and ZIP8 are expressed at both the RNA and protein levels in these cells. To ensure careful analyses, we confirmed that the primers used in our studies are specific to only one target and that the amplicon is the predicted length. In addition, we validated the specificities of anti-ZIP14 and anti-ZIP8 antibodies used in our studies. Rigorous analyses of primers and antibodies are necessary due to the novelty of HIBCPP cells and the relatively recent identification of ZIP14 and ZIP8 as Mn transporters. Since this is the first study to identify ZIP14 and ZIP8 in HIBCPP cells, further studies will greatly benefit from these primer and antibody validations.

The functions of ZIP14 and ZIP8 as Mn importers have been well documented [27,28,30,35,36,37]. Our results confirm that both proteins are functional Mn importers in HIBCPP cells, which is consistent with reports using other epithelial cell lines. We determined the relative subcellular distribution of ZIP14 and ZIP8 to help further understand their potential roles in Mn uptake in HIBCPP cells. We found that HIBCPP cells express ZIP14 primarily at the basolateral membrane and ZIP8 mainly on the apical surface. By understanding the polarized localization of ZIP14 and ZIP8, we can begin to understand how Mn is transported in choroid plexus-derived epithelial cells.

This study has limitations that should be acknowledged. First, the expression of Mn transport proteins in choroid plexus tissue is currently unknown. Further research is needed to determine the in vivo expression patterns of ZIP14 and ZIP8 in the choroid plexus and the function of these two proteins in primary choroid plexus epithelial cells. Additionally, transcriptomic analysis of ZIP transporters in the choroid plexus indicates that transcript levels vary between fetal and neonatal development [57]. While HIBCPP cells originate from an adult, we do not know whether they express the same levels of Mn transporters as healthy human adults. A future investigation will need to analyze the age-dependent expression of ZIP14 and ZIP8 in vivo.

## 5. Conclusions

This report supports two main conclusions that will contribute to the field of Mn biology in the brain. First, we have established a Transwell culture model for HIBCPP cells. These cells form a polarized monolayer with tight junctions and express specific apical and basolateral proteins. Second, we have identified that Mn transporters ZIP14 and ZIP8 both contribute to Mn accumulation by HIBCPP cells, and are enriched on basolateral and apical sides, respectively. Future studies using HIBCPP cells could benefit from our findings to further investigate mechanisms of Mn homeostasis regulation in the choroid plexus-derived cells.

## Supplementary Materials

The following supplemental figures are available online at https://www.mdpi.com/2076-3425/10/8/534/s1, Figure S1: Uncropped Western Blot Images for Figure 2, Figure S2: Uncropped Western Blot Images for Figure 3A, Figure S3: Uncropped Western Blot Images for Figure 5C,D.
