# Supplementary material for: Localization of ZIP14 and ZIP8 in HIBCPP Cells
Source: Brain Sci. 2020 Aug 8;10(8):534. doi: 10.3390/brainsci10080534 (PMC7464652; doi:10.3390/brainsci10080534)
Supplement: Supplementary file 1 [file brainsci-10-00534-s001.pdf]

brain sciences

Supplementary Materials

Localization of ZIP14 and ZIP8 in HIBCPP cells

Shannon E. Morgan[1], Horst Schroten[2], Hiroshi Ishikawa[3] and Ningning Zhao[1*]

[1] Department of Nutritional Sciences, University of Arizona, Tucson, AZ 85721, USA; morgans3@arizona.edu
[2] Department of Pediatrics, Pediatric Infectious Diseases, Medical Faculty Mannheim, Heidelberg University, 68167 Mannheim, Germany; horst.schroten@umm.de
[3] Department of Neurosurgery, University of Tsukuba, Tsukuba, Ibaraki 305-8575, Japan; ishihiro.crm@md.tsukuba.ac.jp
* Correspondence: zhaonn@arizona.edu; Tel.: +1-520-621-9744

List of materials included: Figures S1–S3

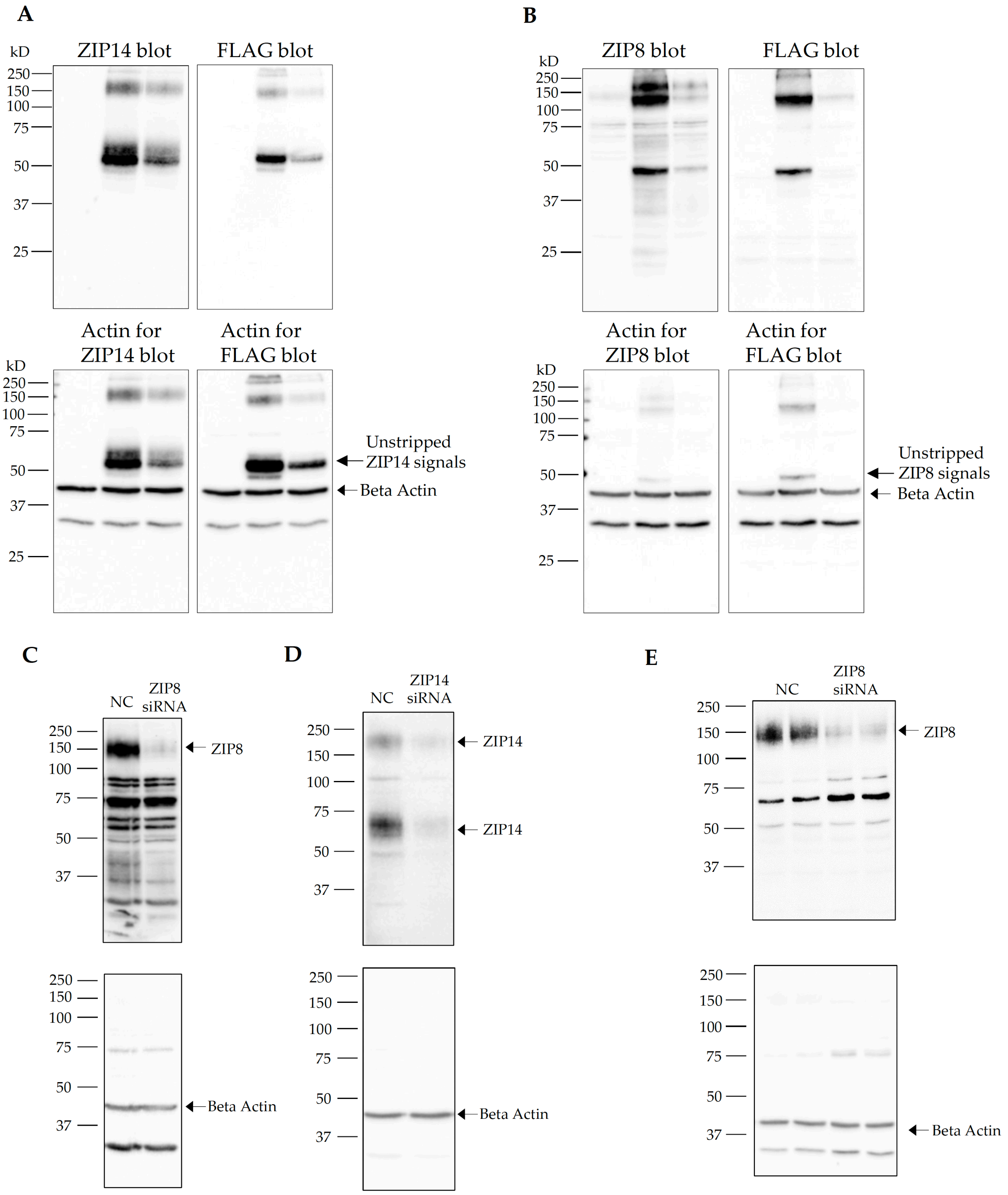

Figure S1. Uncropped immunoblots for Figure 2. (**A**) Full length blots for ZIP14, FLAG, and Beta Actin in Figure 2A. (**B**) Full length blots for ZIP8, FLAG, and Beta Actin in Figure 2B. (**C**) Full length blots for ZIP8 and Beta Actin in Figure 2C. (**D**) Uncropped blots for Figure 2D. (**E**) Uncropped blots for Figure 2E.

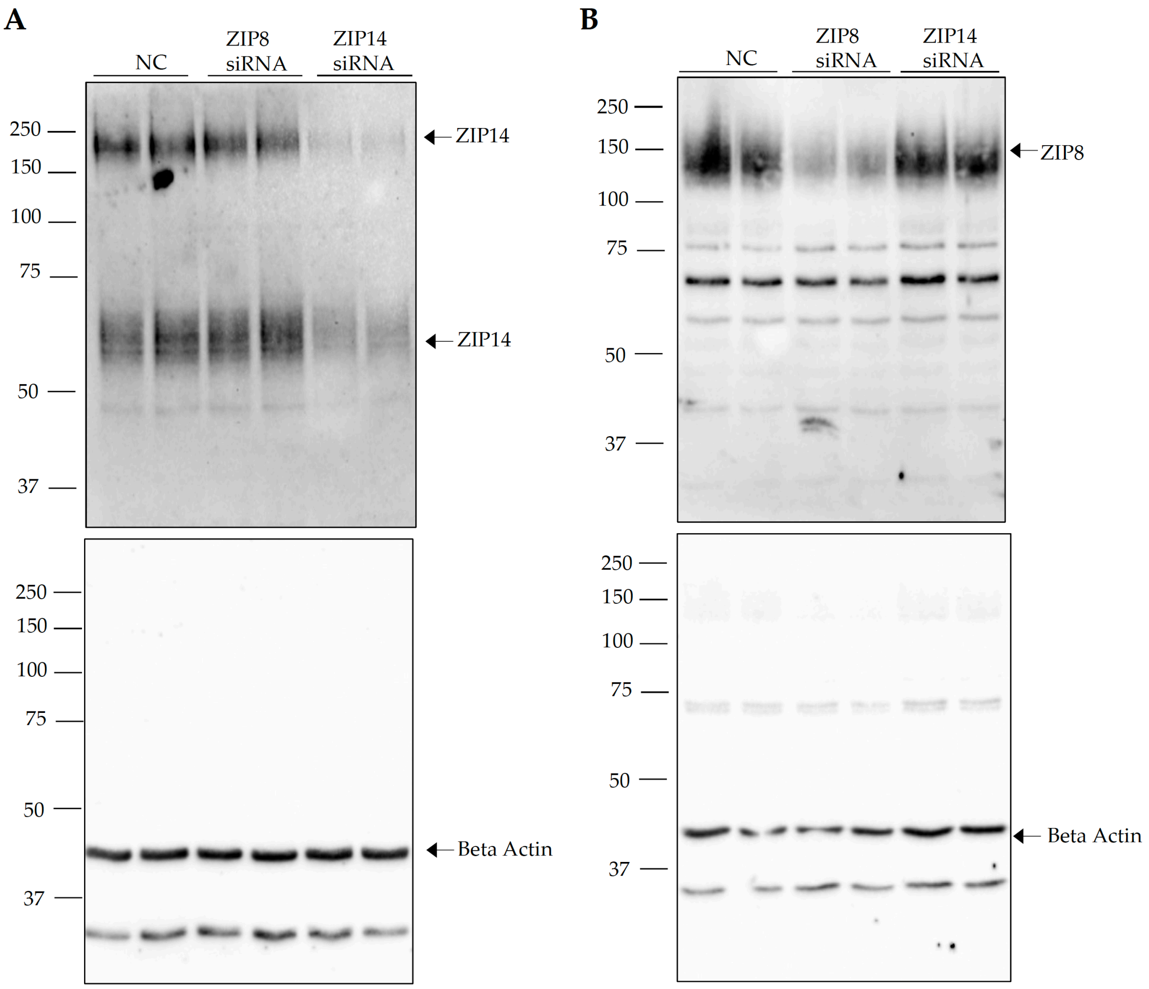

Figure S2. Uncropped immunoblots for Figure 3A. Full length blots for (**A**) ZIP14 and the loading control, and (**B**) ZIP8 and the loading control.

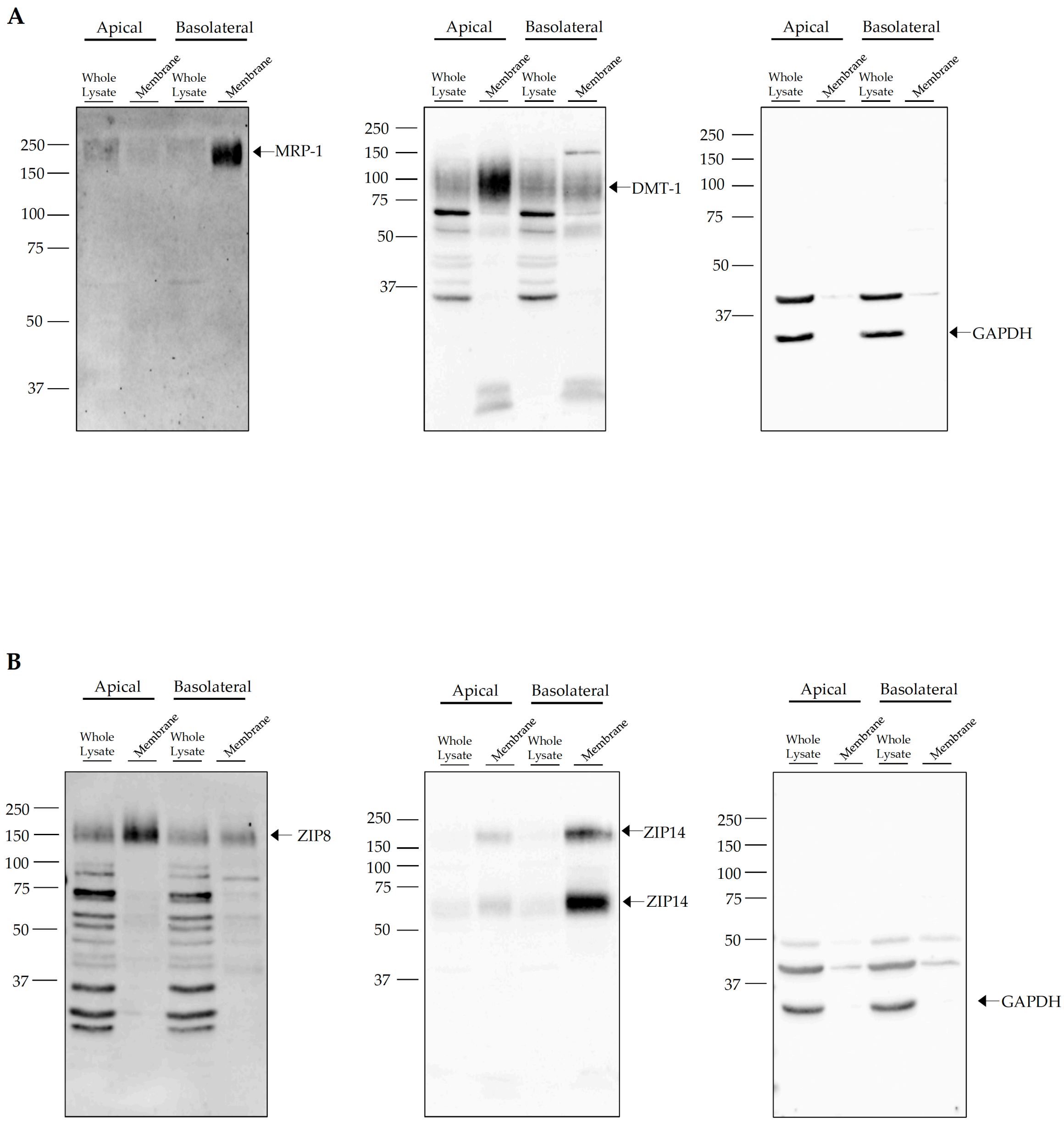

Figure S3. Uncropped immunoblots for Figure 5C and 5D. (**A**) Whole blots for MRP1, DMT1, and GAPDH in Figure 5C. (**B**) Whole blots for ZIP8, ZIP14, and GAPDH in Figure 5D.
